# Central pancreatectomy: A viable option for solid pseudopapillary neoplasm - A case report

**DOI:** 10.1016/j.ijscr.2023.108754

**Published:** 2023-09-01

**Authors:** Aarzish Ijaz, Muhammad Jawad Zahid, Mahnoor Ata Ullah, Musarrat Hussain

**Affiliations:** Resident General Surgery, Hayatabad Medical Complex, Phase 4, Hayatabad, Peshawar, 25000, Pakistan

**Keywords:** Solid pseudopapillary neoplasm, Pancreas, Central pancreatectomy, Female, Prognosis

## Abstract

**Introduction:**

Solid pseudopapillary neoplasms (SPNs) of the pancreas are rare tumors, comprising about 1 % of pancreatic tumors. They primarily affect females during their reproductive phase and have a favorable prognosis. SPNs are usually asymptomatic or present with mild symptoms. The exact histopathogenesis of SPNs remains unknown. Surgical resection is curative, and central pancreatectomy is a pancreas-sparing surgical technique.

**Case presentation:**

A 33-year-old female presented with epigastric pain, vomiting, and infertility. Imaging revealed a mass in the pancreas. Exploration confirmed the mass, and central pancreatectomy was performed. Histopathology confirmed the diagnosis of SPN. The patient's recovery was uneventful, and follow-up CT scans showed no recurrence.

**Discussion:**

This case involves a 33-year-old female presenting with epigastric pain and vomiting, revealing a cystic mass with a solid component in the pancreas. While generally benign, SPNs can become malignant in 15 % cases, with a favorable prognosis. Histopathologically, SPNs remain distinct, with CD99 and CD10 staining confirming the diagnosis. Diagnostic imaging, particularly CT scans, aids in identifying SPNs. Surgical resection, such as central pancreatectomy, is effective, preserving organ function. The case's positive outcome aligns with an overall 5-year survival rate of 95–97 %, highlighting the overall favorable prognosis of SPNs. The procedure's balance between tumor removal and organ preservation offers clinical advantages.

**Conclusion:**

This case underscores the successful management of an SPN using central pancreatectomy. It highlights the importance of early diagnosis and surgical intervention, as well as the favorable prognosis associated with SPNs, even in cases of metastasis. Central pancreatectomy offers organ preservation and reduces long-term complications. Continued reporting and research on such cases contribute to refining treatment strategies for SPNs.

## Highlights

• This report innovates SPN management via central pancreatectomy and pancreaticogastrostomy, showcasing its feasibility.
• Rare SPNs mainly impact young females, stressing early diagnosis and ideal treatment for a promising prognosis.
• A 33-year-old female with epigastric pain revealed a mass at the pancreatic neck-body junction, emphasizing the need for careful evaluation.
• Central pancreatectomy preserves organ function, minimizes pancreatic insufficiency risk.
• Central pancreatectomy's success and recurrence-free follow-ups highlight its efficacy in managing SPNs

## 1 Introduction

Solid pseudopapillary neoplasms (SPN) of the pancreas, first reported by Frantz in 1959, are rare clinical entities, accounting for approximately 1 % of all pancreatic tumors. [1] These tumors mostly affect females during their reproductive age, and fortunately, they have a good prognosis. [2] SPNs are primarily located in the body or tail of the pancreas and tend to be asymptomatic or present with mild symptoms, such as epigastric discomfort, nausea, and dyspepsia. [3] Despite their prevalence, the histopathogenesis of SPNs remains unknown. [4] To diagnose SPNs, various imaging modalities, including ultrasonography (US), computed tomography (CT), and magnetic resonance imaging (MRI), can differentiate them from other pancreatic lesions. Surgical resection of SPNs is curative and offers long-term survival. [5] For benign or low-grade malignant lesions affecting the pancreatic body and neck, central pancreatectomy, a pancreas-sparing technique, has emerged as the alternative surgical option. [6] This approach enables organ preservation while avoiding exocrine and endocrine pancreatic insufficiency. [7] This case has been reported in line with SCARE criteria. [8]

## 2 Case presentation

A 33-year-old female presented with epigastric pain that extends to both sides, which occurred 3 to 4 h after eating. She also experienced occasional non-projectile vomiting with partially digested food. Additionally, she had a history of oligomenorrhea and primary infertility for the past 12 years. There were no documented instances of weight loss. Her medical history included a past typhoid fever case but no previous surgeries.

**Fig. 1:**
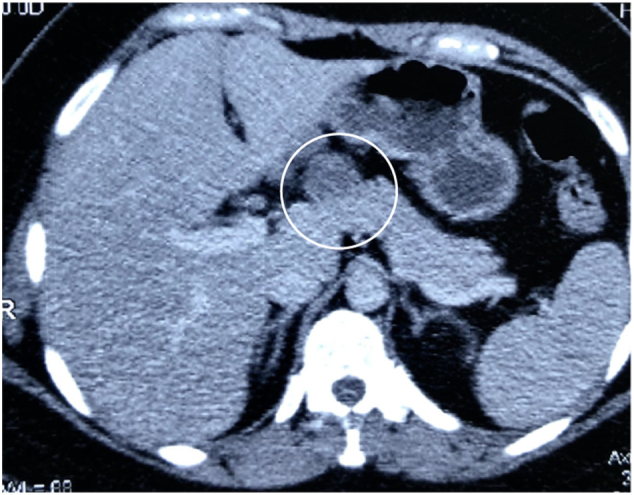
CT scan showing a solid mass at the junction of the neck and body of the pancreas. The mass is encircled.

Upon evaluation, an ultrasonographic scan of her abdomen and pelvis confirmed the presence of cholelithiasis. She was scheduled for a laparoscopic cholecystectomy at a different hospital. Before the procedure, a repeat ultrasonographic scan was performed to confirm the cholelithiasis and examine the pancreas. The ultrasound showed a 27 * 32 mm hypoechoic lesion in relation to the body of the pancreas, while the head and tail were normal after which a CT scan was ordered. The CT scan confirmed these findings, and an EUS showed a well-defined, rounded cystic mass with a solid component at the junction of the head and body of the pancreas. (See Fig. 1) Multiple EUS guided biopsies were taken, and histopathology report showed a papillary structure with central fibrovascular core and lined by mononuclear cells with impression of solid pseudopapillary neoplasm of pancreas.

Subsequently, the patient was referred to a tertiary care setting. The necessary blood tests and examinations were carried out. On upper abdominal palpation, mild tenderness was noted in the epigastric region. Other aspects of the physical examination appeared normal. Her hemoglobin level was 12.3 g/dl, and her T. bilirubin, SGPT, and ALP levels were within normal ranges. Renal function tests (RFTs) and coagulation profiles were also normal, and virology results were negative. Based on the above findings and a negative metastatic workup, she agreed to undergo exploration. As a result, she underwent elective exploration using a median approach.

**Fig. 2:**
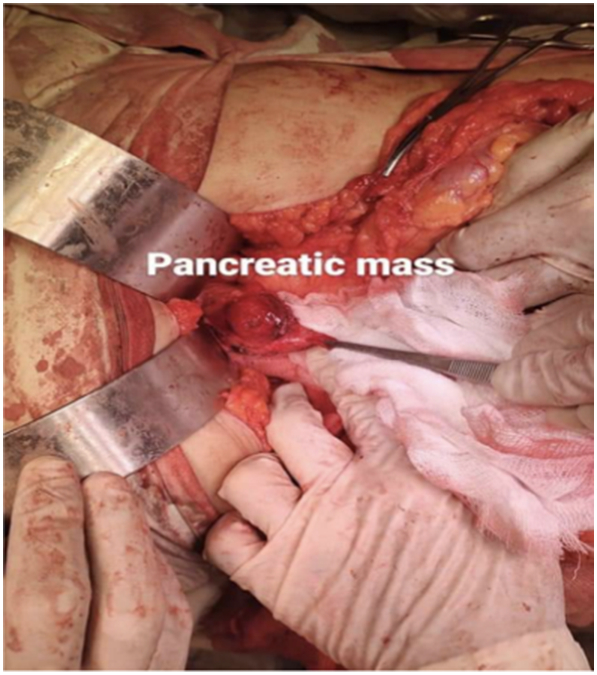
Pancreatic mass at the junction of its neck and body.

**Fig. 3:**
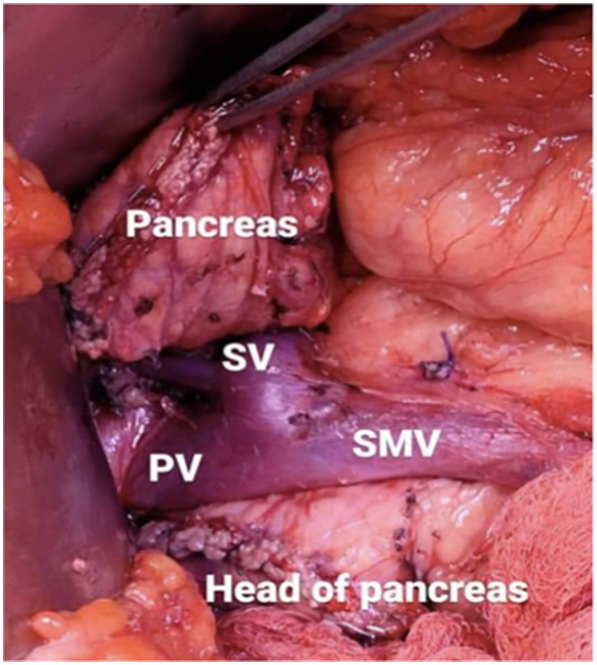
Post-mass resection with preserved vasculature and remnant pancreatic body, tail, and head.

During the exploration, a mass was discovered at the junction of the pancreatic neck and body. (See Fig. 2) In response, a pancreas-preserving procedure known as a central pancreatectomy was performed. An anastomosis was established between the distal pancreatic stump and the posterior wall of the stomach through pancreaticogastrostomy, with careful attention to avoid damaging the SMV, SMA, splenic vein, and portal vein. (See Fig. 3).

The resected specimen was sent for histopathological examination, which confirmed the preoperative diagnosis of a solid pseudopapillary tumor of the pancreas. The surgical margins were free of tumors, and immunohistochemistry showed dot-like positivity for CD99 and overall positivity for CD10.

The postoperative period was uneventful, with the patient resuming oral intake shortly after the surgery. Regular serum blood glucose monitoring and early mobilization were conducted, and no immediate complications arose. She was discharged from the hospital after a week.

**Fig. 4:**
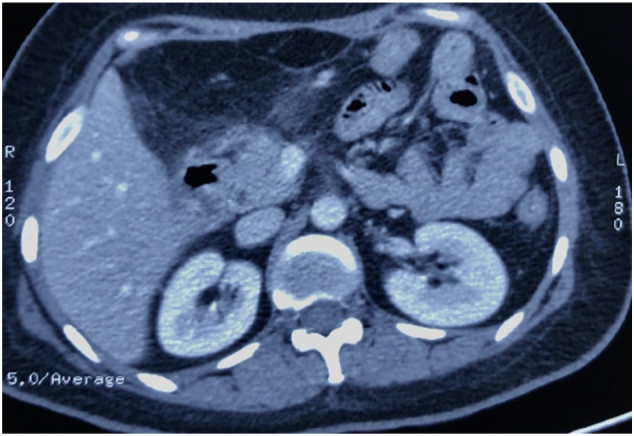
CT scan at 1-year follow-up showing complete resolution of the tumor.

The patient attended follow-up visits and underwent CT scans. The most recent CT scan revealed a normal pancreatic body, uncinate process, and tail. The pancreatic duct was draining into the posterior wall of the stomach body, with no evidence of a mass at the anastomosis site, and the anastomosis remained intact. (See Fig. 4).

## 3 Discussion

Solid pseudopapillary neoplasms of the pancreas are extremely rare, constituting approximately 1–6 % of all pancreatic tumors. Initially referred to as “benign or malignant papillary neoplasms of the pancreas,” these lesions have been designated by various names, including solid and cystic lesions of the pancreas, papillary cystic lesions, and Frantz tumors. [9] In 1996, the World Health Organization (WHO) standardized the nomenclature as “Pseudopapillary Neoplasms of the Pancreas.” The exact origin of SPT remains uncertain, with some researchers suggesting a pluripotent cell origin while others favor an extrahepatic origin from genital ridge angle-related cells. These tumors predominantly affect young women, with a male-to-female ratio of 1:10. [5] In this case, the patient is a 33-year-old female. Although most SPTs are benign, malignancy can arise in about 15 % of cases. This may involve adjacent structures or result in distant metastases, with the liver and omentum being the most common sites of metastasis. Despite their locally aggressive characteristics, SPTs generally have an excellent prognosis due to their low malignant potential. [3]

SPTs typically present with non-specific symptoms, and they are often discovered incidentally. Some common symptoms include abdominal pain, nausea, vomiting, dyspepsia, weight loss, and the presence of a slowly growing abdominal mass. In some cases, they can also manifest as jaundice, pruritus, asthenia, back pain, and pancreatitis. [8] In this specific case, the patient experienced epigastric pain that extends to both sides, accompanied by episodes of nausea and vomiting. Imaging studies usually reveal SPTs as large cysts with heterogeneous solid and cystic components. CT scans are the preferred diagnostic modality, while MRI is useful for differentiating between solid and cystic elements. Fine Needle Aspiration Cytology (FNAC) can aid in the diagnosis, but it carries risks of complications like neoplastic cell seepage and the formation of pancreatic biliary fistulas. Hence, FNAC should only be performed when a clear diagnosis cannot be made through radiological evidence. [2]

SPTs are known for their characteristic features and can occur in various locations within the pancreas, though they are most frequently found in the head or tail. [[9], [10], [11], [12], [13]] In the presented case, an abdominal ultrasound revealed a 27 * 32 mm hypoechoic lesion in relation to the body of the pancreas, while the head and tail were normal. A subsequent CECT confirmed these findings, and an EUS showed a well-defined, rounded cystic mass with a solid component at the junction of the head and body of the pancreas. Histologically, SPNs are characteristically positive for α1-antitrypsin, CD56, CD10, and vimentin. [3,5,14] Multiple biopsies were taken and examined histopathologically, confirming it as an SPT. The tumor in this case was positive for CD99 and CD10.

The primary treatment for SPNs is complete or radical resection, even in cases with advanced disease and metastasis. [12] Organ preservation is essential, with distal pancreatectomy with spleen preservation being the preferred procedure for tumors in the body and tail of the pancreas. Tumors in the neck of the pancreas are better treated with central pancreatectomy along with distal pancreaticojejunostomy or pancreaticogastrostomy. [15] When considering a patient for central pancreatectomy, key factors include confirming a benign or borderline lesion through surgery, favoring lesions in the pancreas neck or proximal body, avoiding enucleation near the pancreatic duct, and ensuring a distal pancreatic stump of at least 6 cm for preservation. It allows for the preservation of normal tissue, reducing long-term morbidity and complications such as diabetes mellitus and pancreas gland insufficiency while maintaining high oncological efficiency. [6,7] However, central pancreatectomy with pancreaticojejunostomy can lead to complications such as pancreatic fistulas, enteric fistulas, or anastomotic stump leaks. [15,16]

In this specific case, the patient underwent a central pancreatectomy with pancreaticogastrostomy for the mass located at the junction of the neck and body of the pancreas. Fortunately, SPTs have a favorable prognosis, even in cases of metastasis and local recurrence, with an overall 5-year survival rate of 95–97 %. Local recurrence occurs in less than 10 % of cases and usually happens around 4 years after surgery. [12,17] Postoperatively, the patient, in this case, experienced an uneventful recovery, and a follow-up CT scan at 1 year's post-surgery did not reveal any signs of recurrence or residual disease.

## 4 Conclusion

In conclusion, this case report highlights the successful management of a rare solid pseudopapillary neoplasm (SPN) of the pancreas using central pancreatectomy with pancreaticogastrostomy. SPNs, though rare, predominantly affect young females and can present with non-specific symptoms. Early diagnosis and prompt surgical intervention are essential for achieving favorable outcomes. Central pancreatectomy offers the advantage of organ preservation while maintaining high oncological efficacy and reducing long-term complications. Overall, the patient had an uneventful postoperative period and remained free from recurrence during follow-up visits. This reinforces the generally excellent prognosis associated with SPNs. Continual reporting and research on such cases will contribute to a better understanding of SPNs and aid in refining treatment strategies for optimal patient care. The success of central pancreatectomy in this case underscores its viability as a preferred surgical option for selected SPNs, providing valuable insights for future clinical management.

## Sources of funding

N/A

## Consent

Written informed consent was obtained from the patient for the publication of this case report and accompanying images. A copy of the written consent is available for review by the Editor-in-Chief of this journal on request.

## Registration of research studies

N/A.

## Provenance and peer review

Not commissioned, externally peer reviewed.

## Declaration of competing interest

There was no conflict of interest to declare.
